# A Carotenoid- and Poly-β-Hydroxybutyrate-Free Mutant Strain of *Sphingomonas elodea* ATCC 31461 for the Commercial Production of Gellan

**DOI:** 10.1128/mSphere.00668-19

**Published:** 2019-10-16

**Authors:** Ang Li, Tingting Hu, Hangqi Luo, Nafee-Ul Alam, Jiaqi Xin, Hongwei Li, Yinuo Lin, Jingyu Huang, Ke Huang, Yuan Meng, Fenbin Meng, Xiufang Hu, Ou Li

**Affiliations:** a College of Life Science and Medicine, Zhejiang Sci-Tech University, Hangzhou, China; Martin Luther University of Halle-Wittenberg Institute of Biology/Microbiology

**Keywords:** gellan, poly-β-hydroxybutyrate, gene knockout, mutagenesis, carotenoids

## Abstract

Gellan gum is a microbial exopolysaccharide, produced after aerobic fermentation using the Gram-negative bacterium strain Sphingomonas elodea ATCC 31461. Due to its unique structure and excellent physical characteristics, gellan gum has a broad range of applications in food, pharmaceutical, and other industries where it is used for stabilizing, emulsifying, thickening, and suspending. During the fermentative production of gellan, strain ATCC 31461 also accumulates large amounts of the metabolic by-products yellow carotenoid pigments and poly-β-hydroxybutyrate (PHB), which is decreasing the gellan production and increasing processing costs. A pigment PHB-free mutant was obtained by knocking out the phytoene desaturase gene (*crtI*) in the carotenoid biosynthetic pathway and the *phaC* gene, encoding a PHB synthase for the polymerization of PHB. Unfortunately, the double gene knockout mutant produced only 0.56 g liter^−1^ gellan. Furthermore, blocking PHB and carotenoid synthesis resulted in the accumulation of pyruvate, which reduced gellan production. To elevate gellan production, combined UV irradiation and ethyl methanesulfonate (EMS) mutagenesis treatment were used. A mutant strain with the same level of pyruvate as that of the wild-type strain and higher gellan production was isolated (1.35 g liter^−1^, 132.8% higher than the double gene knockout mutant and 14.4% higher than the wild-type strain ATCC 31461). In addition, a new gellan gum recovery method based on the new mutant strain was investigated, in which only 30% isopropanol was required, which is twice for the wild-type strains, and the performance of the final product was improved. Thus, the mutant strain could be an ideal strain for the commercial production of gellan.

**IMPORTANCE** A carotenoid- and PHB-free double gene knockout strain mutant was constructed to simplify the purification steps normally involved in gellan production. However, the production of gellan gum was unexpectedly reduced. A mutant with 14.4% higher gellan production than that of the wild-type strain was obtained and isolated after employing UV and EMS combined mutagenesis. Based on this high-yield and low-impurity-producing mutant, a new recovery method requiring less organic solvent and fewer operating steps was developed. This method will effectively reduce the production costs and improve the economic benefits of large-scale gellan production.

## Abstract (precis)

A carotenoid- and PHB-free double gene knockout strain mutant was constructed to simplify the purification steps normally involved in gellan production. However, the production of gellan gum was unexpectedly reduced. A mutant with 14.4% higher gellan production than that of the wild-type strain was obtained and isolated after employing UV and EMS combined mutagenesis. Based on this high-yield and low-impurity-producing mutant, a new recovery method requiring less organic solvent and fewer operating steps was developed. This method will effectively reduce the production costs and improve the economic benefits of large-scale gellan production.

## INTRODUCTION

Microbial exopolysaccharides as industrial products of microbial fermentation not only have excellent application performance but also have wider applications than plant and animal polysaccharides do. These polysaccharides are not constrained by geographical, environmental, climatic, and other factors. In addition, a short production cycle, stable yield, and good quality, as well as a high performance-price ratio, render them largely able to meet the demands for natural, pollution-free food and other applications. Gellan gum is a novel microbial exopolysaccharide, produced after aerobic fermentation using the Gram-negative bacterium strain Sphingomonas elodea ATCC 31461 (1). This linear heteropolysaccharide, with an average molecular mass of 0.5 MDa, is based upon an acetylated tetrasaccharide repeating unit consisting of d-glucose, l-rhamnose, and d-glucuronic acid at a ratio of 2:1:1 (2). At present, the global market demand for gellan is about 10,000 tons, and as a new microbial polysaccharide, demand is still growing. Furthermore, its potential use as an alternative to gelatin and agar makes it the most significant commercialized bacterial exopolysaccharide (3). Market demand for gellan has been increasing year by year, with an annual growth rate of more than 30% (4, 5). Since the raw material (such as sucrose) needed for production of gellan is inexpensive and plentiful, but gellan has a very high market price ($17 to 21/kg), there are extremely high commercial profit and good market prospects. Although gellan gum exhibits outstanding properties, two major metabolic by-products produced hinder the development of the gellan industry. In the food and cosmetic industries, two major metabolic by-products affect the transparency of products that can directly affect the acceptability to consumers. First, Sphingomonas elodea synthesizes a unique yellow carotenoid product that renders the fermentation broth a yellow color. In addition to the yellow pigment, the high-carbon, low-nitrogen conditions needed for the production of gellan polysaccharide can also be conducive to the synthesis of another insoluble impure poly-β-hydroxybutyrate (PHB).

PHB is a significant contributor to the turbidity exhibited by reconstituted gellan gum solutions. The market needs the more transparent products for practical applications, but the yellow carotenoid and PHB are the major deterrents to an easily achieved clarification process. The high viscosity of the fermentation broth at the end of the process makes it difficult to separate gellan from the broth (6). The general procedure for recovering the biopolymer requires 2 volumes of ethanol or isopropyl ethanol for removing pigment, as well as precipitating polysaccharides, followed by removal of PHB with a diatomite plate and frame filter. This extraction method necessitates large amounts of ethanol, greatly increasing the cost of production. Second, carotenoid and PHB share the common precursor gellan (7, 8). As a result, the gellan production is partly reduced due to its competition for the limited carbon sources.

In this study, a pigment PHB-free mutant was obtained by knocking out the phytoene desaturase gene (*crtI*) in the carotenoid biosynthetic pathway and the *phaC* gene, encoding a PHB synthase for the polymerization of PHB. Unfortunately, the double gene knockout mutant produced less gellan. To elevate gellan production, combined UV irradiation and ethyl methanesulfonate (EMS) mutagenesis treatment were used. In addition, a new gellan gum recovery method based on the new mutant strain was investigated. Thus, the mutant strain could be an ideal strain for the commercial production of gellan.

## RESULTS

### Construction of Δ*crtI* mutant strain.

The pLO_3_ plasmid could be replicated in Escherichia coli, which contains the λPIR protein, while it could not be replicated independently in Sphingomonas elodea. Therefore, when the pLO_3_ plasmid, as a suicide vector, entered the wild-type strain with the assistance of a helper plasmid, it must have integrated into the genome chromosome; otherwise, the plasmid would be lost (see Fig. S1 in the supplemental material).

10.1128/mSphere.00668-19.1 FIG S1 Schematic of two rounds of single crossover homologous recombination for gene knockout illustrating the steps used to clone and construct an internal deletion in the *Sphingomonas elodea crtI* gene. The flanking regions (upstream and downstream sequences) of the nucleotides of interest (*crtI* for this work) were cloned to the “suicide” and integration vector pLO_3_, which can replicate in a host containing λpir protein suitable for plasmid construction, such as E. coli, but cannot replicate in *Sphingomonas*. Two rounds of single crossover events were used to eliminate the *crtI* gene in *S. elodea*. For the first round of single crossover, selection in *Sphingomonas* for tetracycline (tet) resistance encoded by the plasmid identifies these colonies in which the whole plasmid has integrated into the chromosome as a result of homologous recombination. For the second round of single crossover, selection for the loss of antibiotic resistance and resistance to high-concentration sucrose plate identified colonies in which the duplicated region has recombined out, which may result in two types: intact or mutant strains of a deletion of the *crtI* gene. Download FIG S1, TIF file, 0.4 MB. Copyright © 2019 Li et al. 2019 Li et al. This content is distributed under the terms of the Creative Commons Attribution 4.0 International license.

**FIG 1:**
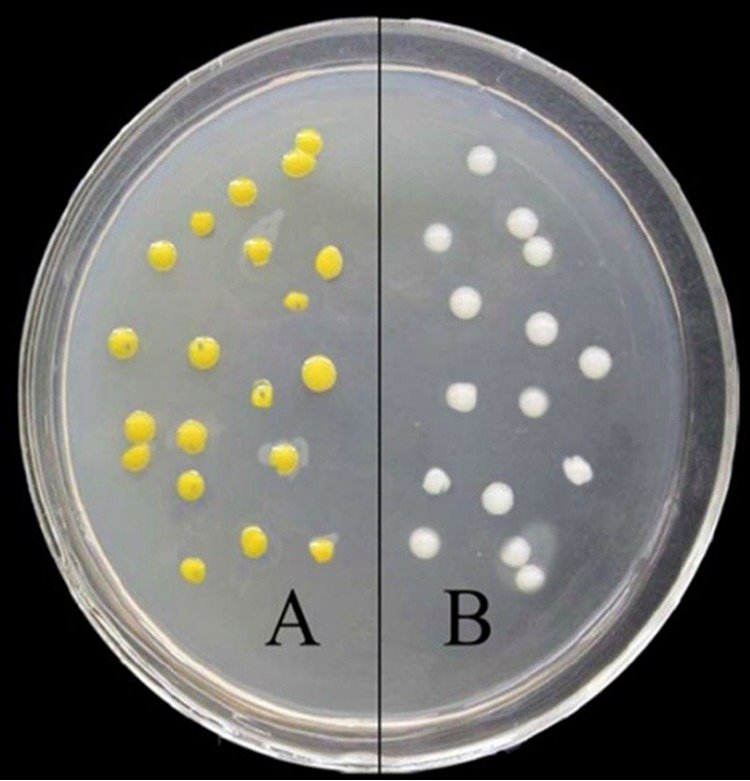
Phenotype of prototrophic strain (A) and *crtI* gene knockout strain (B) on a plate. The cells were incubated on YM agar plates at 30°C for 48 h. Wild-type strains were yellow, while the *crtI* gene knockout strains were white.

After two rounds of homologous recombination exchange, seven single colorless colonies were selected randomly after the LB liquid culture extracted the bacterial genomic DNA. Agarose gel electrophoresis was performed after PCR amplification. The *crtI* gene was knocked out, and the mutants were confirmed by PCR and DNA sequencing (Fig. S2). Compared to the yellow-pigmented wild-type strain, the Δ*crtI* mutants were colorless (Fig. 1). Furthermore, the wild-type strain exhibited several yellow carotenoid pigments when analyzed by HPLC (high-performance liquid chromatography), while no pigments could be found for the Δ*crtI* mutants. Gellan production measurements showed there was no significant difference between the wild type and the Δ*crtI* mutant.

10.1128/mSphere.00668-19.2 FIG S2 Two rounds of homologous crossover and the phenotype of prototrophic strain. Lane 1 contains the positive strain of the first round of homologous crossover tested by PCR. Primers (*sac*B-F and *sac*B-R) were designed to amplify a 450-bp fragment of the *sacB* gene. The wild-type strain (lane 2) and *crtI* gene knockout strain (lane 3) of the second round of homologous crossover were tested by PCR. (The primers were *crt*I-TF [5′-GTCTATTGCCTGCCGTTC--3′] and *crt*I-TR [5′-GGCTGATAGCGTGTTTTC--3′].) Download FIG S2, TIF file, 0.3 MB. Copyright © 2019 Li et al. 2019 Li et al. This content is distributed under the terms of the Creative Commons Attribution 4.0 International license.

### Construction of Δ*crtI*-Δ*phaC* double gene knockout mutants and determination of PHB production and gellan production.

To construct Δ*crtI*-Δ*phaC* double gene knockout mutants, the Δ*crtI* mutant was used as the original strain. The double gene knockout mutants were confirmed by PCR and DNA sequencing (Fig. S3). PHB content measurement showed very low values for all the Δ*phaC* mutants, which indicated that the Δ*phaC* knockout strains did not produce PHB, while the wild-type strain produced 3.15 g liter^−1^ PHB particles. The gellan production results are shown in Table 1. Compared with the control group, the viscosity and yield of gellan for the Δ*phaC*-Δ*crtI* gene knockout strains were significantly decreased, and the highest gellan production of the recombinant strains was only 46.7% of the control group. These results were consistent with those obtained by Baird and Cleary (9).

**TABLE 1:** Determination of the fermentation performance of Sphingomonas elodea Δ*phaC*-Δ*crtI* mutants

Strain	Viscosity (cP)^*a*^	Gellan production (g liter^−1^)^*a*^
ATCC 31461	5,267 ± 287	1.20 ± 0.15
Δ*phaC*-Δ*crtI*-1	833.3 ± 105	0.48 ± 0.08
Δ*phaC*-Δ*crtI*-2	1,633 ± 138	0.56 ± 0.09
Δ*phaC*-Δ*crtI*-3	1,500 ± 201	0.55 ± 0.11
Δ*phaC*-Δ*crtI*-4	1,550 ± 107	0.50 ± 0.11
Δ*phaC*-Δ*crtI*-5	1,683 ± 88	0.47 ± 0.13
Δ*phaC*-Δ*crtI*-6	1,650 ± 160	0.46 ± 0.07
Δ*phaC*-Δ*crtI*-7	1,300 ± 152	0.42 ± 0.10

a Means of triplicate measurements. Values were significantly different (*P* < 0.05) based on analysis of variance (ANOVA) and Tukey’s tests.

10.1128/mSphere.00668-19.3 FIG S3 Lanes 1 to 24 were some of the randomly selected single colonies after two rounds of crossover deletion. The short electrophoretic strip of the lanes 4, 5, 11, 12, 14, 16, and 22 were the samples whose *phaC* genes were successfully knocked out. Download FIG S3, TIF file, 0.6 MB. Copyright © 2019 Li et al. 2019 Li et al. This content is distributed under the terms of the Creative Commons Attribution 4.0 International license.

### Pyruvate content determination.

**FIG 2:**
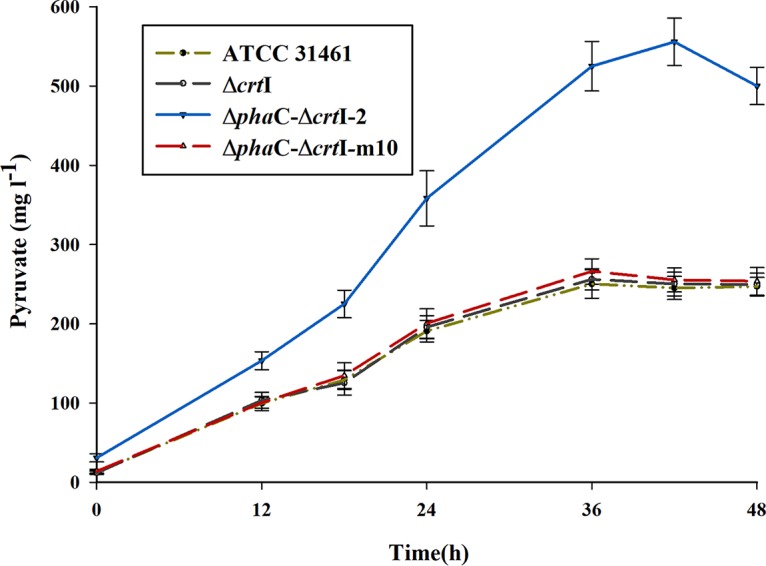
Pyruvate content over time for four strains. The strains tested were the original production strain ATCC 31461, the single gene knockout strain Δ*crtI* strain, the double gene knockout strain Δ*phaC*-Δ*crtI*-2, and the mutation screening strain of the double gene knockout strain, the Δ*phaC*-Δ*crtI*−m10 strain. Pyruvate content is given in milligrams liter^−1^. The pyruvate content for the Δ*pha*C-Δ*crt*I−m10 strain was close to that of the original strain.

To analyze the reasons why the Δ*phaC*-Δ*crtI* double gene knockout mutant was associated with lower production of gellan gum, the time courses of the pyruvate content in the broths of the different strains are compared in Fig. 2. Generally, the pyruvate can be exported out of the cell through the cotransport of hydrogen ions with a special proton pump. In the initial phase of fermentation, the pyruvate contents of these strains increased dramatically and reached their peaks at 36 h or 42 h (for the Δ*phaC*-Δ*crtI*-2 mutant), followed by small decreases until 48 h, because of the decrease in the metabolic rate. The results showed that there was no significant difference in pyruvate contents between the ATCC 31461 strain and Δ*crtI* mutant and that the Δ*phaC*-Δ*crtI*-2 mutant had noticeably higher pyruvate content compared with ATCC 31461 and Δ*crtI* mutant at each period of analysis.

### Mutagenesis screening of the Δ*phaC*-Δ*crtI* strain.

To restore or even elevate the gellan production for the double gene knockout strains, the Δ*crtI*-*phaC*-2 mutant was used as the parent strain for UV and EMS combined mutagenesis. By completing three independent mutagenesis experiments, a total of 15,000 mutations were produced, and the survival rate of the strains carrying these mutations was 1%. Among the mutant strains, a total of 358 mutant strains whose colonies were larger and more mucoid than that of the parent strain were screened in this experiment. One of the mutant strains, namely, the Δ*phaC*-Δ*crtI*–m10 mutant, whose yield of gellan was 1.35 g liter^−1^, exhibited increased gellan production of 132.8% compared to that of its parent Δ*crtI*-*phaC*-2 strain (0.56 g liter^−1^), which was 14.4% higher than the wild-type strain ATCC 31461 (1.18 g liter^−1^). The pyruvate content results show that organic acid content also decreased to almost the same as that of the wild-type strain (Fig. 2). A continuous passage experiment indicated that this Δ*phaC*-Δ*crtI*–m10 mutant was stable after 20 continuous passages.

### Analysis of sucrose consumption, gellan production, and cell growth in batch cultivation.

**FIG 3:**
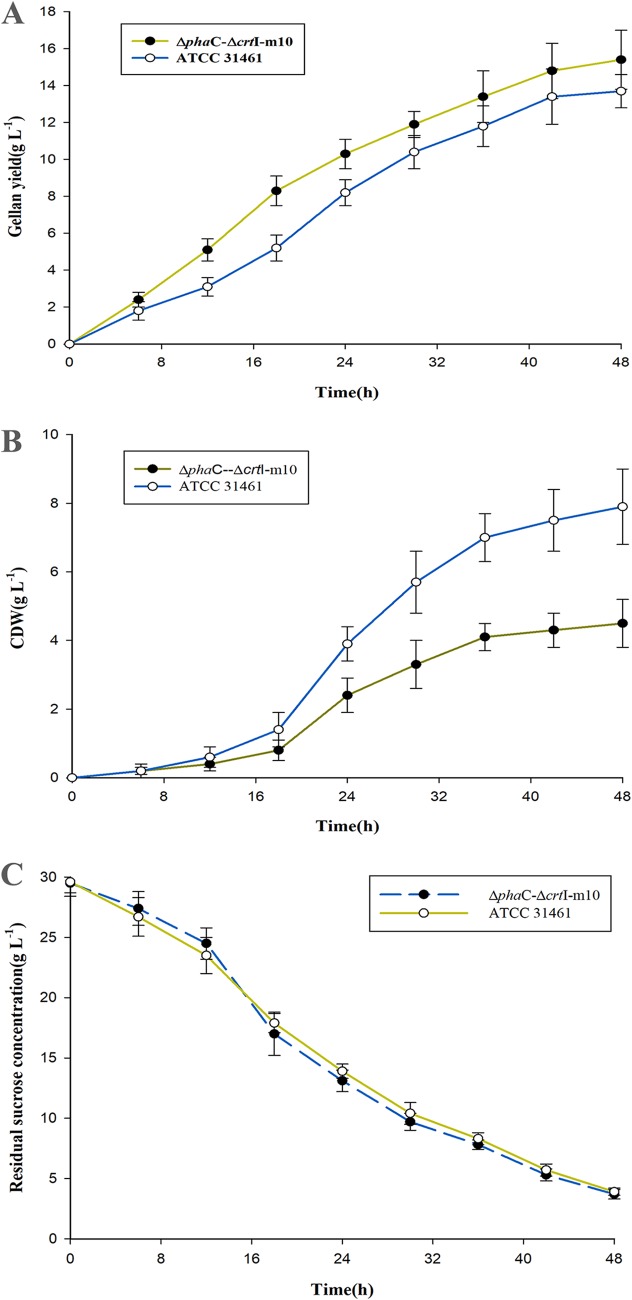
Gellan gum yield (A), cell dry weight (B), and the residual sucrose concentration (C) over time for the double gene knockout and mutagenesis screening strain Δ*phaC*-Δ*crtI*–m10 compared with the original production strain ATCC 31461. (A) The gellan production of the mutant strain was consistently higher than that of the parent strain over the same periods. (B) There was an obvious decrease in cell dry weight (CDW) compared with the original production strain ATCC 31461. (C) The sucrose consumption rates for the Δ*phaC*-Δ*crtI*–m10 mutant and the ATCC 31461 strain were similar over the same period of fermentation.

To further evaluate and compare the wild-type and Δ*phaC*-Δ*crtI*–m10 mutant strains, batch fermentation experiments were conducted in bioreactors. Gellan is a primary metabolite that was produced with the growth of bacteria. Large amounts of gellan were produced during the logarithmic growth phase. After 48 h of cultivation, the mutant was found to achieve higher gellan levels than those produced by the ATCC 31461 strain throughout the whole fermentation process. The final gellan level accumulated by the mutant strain was up to 15.43 g liter^−1^, 13% higher than the level produced by the wild-type strain (Fig. 3A). The cell dry weight (CDW) reached the maximum level at 36 h and increased little while gellan was constantly produced. There was an obvious decrease in CDW (2.78 ± 0.12 g liter^−1^ for the Δ*phaC*-Δ*crtI*–m10 mutant compared with 4.12 ± 0.16 g liter^−1^ of the wild-type strain) in Fig. 3B. Furthermore, the residual sucrose concentrations for the Δ*phaC*-Δ*crtI*–m10 mutant and ATCC 31461 were similar over the same period of fermentation (Fig. 3C).

### Effects of mutant strain-based recovery method.

Based on the carotenoid-PHB-free mutant strain broth, a new recovery method was developed. To remove pigment and PHB, twice the volume of broth of ethanol and a plate frame pressure filtration were needed. Alternatively, the mutant strain-based recovery method merely consumed 10 g liter^−1^ CaCl_2_ and 30% ethanol (vol/vol) to flocculate the gellan gum. Through this new method, the recovery yield of gellan gum obtained from the mutant was 94.2%, which was close to that from ATCC 3146, which was recovered via the conventional method (94.5%). At the same time, the final product had improved color and light transmittance (Table 2).

**TABLE 2:** Comparison of two gellan recovery methods^*a*^

Strain		Method		Consumption of isopropanol (vol/vol)		Residual carotenoid content (mg g liter^−1^)		Transmittance (%)		Recovery yield of gellan (%)	
Δ*phaC*-Δ*crtI*−m10		CaCl_2_		30%		No		90		94.2	
ATCC 31461		Isopropanol		2 times		0.07		85		94.5	

a All experimental data were means of triplicate measurements.

## DISCUSSION

**FIG 4:**
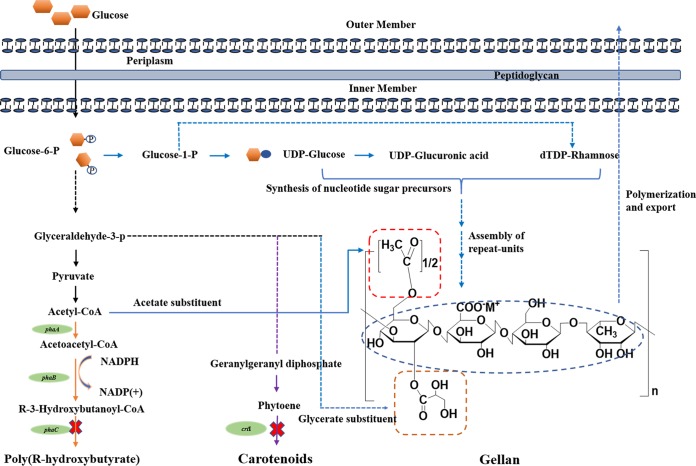
Schematic diagram of PHB, carotenoid, and gellan production in *Sphingomonas elodea* ATCC 31461. The common synthetic pathway is shown on the black arrow. The pathway for gellan biosynthesis includes the synthesis of the three-nucleotide sugar precursors, assembly of the repeat unit by specific glycosyltransferases, polymerization, and export (blue arrow). The pathway for PHB biosynthesis starts from acetyl-CoA (orange arrow), which is also the precursor of acetate substituent of gellan. The pathway for carotenoid biosynthesis starts from glyceraldehyde-3-phosphate (glyceraldehyde-3-p) (orange arrow), which is also the precursor of acetyl-CoA and glycerate substituent of gellan.

In this work, the PHB biosynthetic gene *phaC* was also deleted in the *crtI* background. While gellan production by the *crtI* mutant was similar to that of the wild-type strain, the double knockout mutant showed a significant decrease in production of the polysaccharide (Table 1). Carotenoids are synthesized metabolically via an initial condensation reaction between pyruvate and glyceraldehyde-3-phosphate (G3P) (10), which could be transformed into acetyl coenzyme A (acetyl-CoA) (Fig. 4) (11) and the acylation substituent of gellan (12). Therefore, based on reasonable speculation, the Δ*phaC*-Δ*crtI* double gene knockout mutant should have produced more gellan gum because there should be more carbon sources available for the synthesis of gellan. However, in actuality, the results were contrary to predictions. To analyze the reasons for this confusing and unexpected result, the time courses of the pyruvate content in the broths of different strains were compared in Fig. 2. The reason why there was no significant difference in pyruvate contents and gellan production between the ATCC 31461 strain and Δ*crtI* mutant might be due to the low concentration of carotenoid produced by ATCC 31461, with the total content being about 15 mg liter^−1^. However, PHB could be present in amounts of 3.15 g liter^−1^. As a consequence, the block of PHB in the Δ*phaC*-Δ*crtI*-2 mutant might affect the whole glucose metabolic network and result in the accumulation of metabolic intermediates (including organic acid such as pyruvate). According to the previous reports, the accumulation of organic acids might have an adverse effect on gellan production (13, 14). In addition, some of the enzymes in the carotenoid and PHB synthetic pathways are NADP(H)-dependent reactions. Wu et al. have reported that the block of PHB in Sphingomonas sanxanigenens NX02 may break the balance of NADPH and NADP^+^ of the whole metabolic network, resulting in the decrease of exopolysaccharide (EPS) (15).

To elevate gellan production, combined UV irradiation and EMS mutagenesis treatment were used. A mutant strain producing the same level of pyruvate as the wild type and producing more gellan was isolated (1.35 g liter^−1^, 132.8% higher than the double gene knockout mutant and 14.4% higher than the wild-type strain). These results further proved that the imbalance of the whole glucose metabolic network may result in the accumulation of the metabolic intermediates (including organic acids such as pyruvate) and finally be harmful to gellan production.

To further evaluate the performance of the Δ*phaC*-Δ*crtI*–m10 mutant in more stable conditions, batch fermentation was carried out in a 6.7-liter bioreactor. Sucrose consumption rates for both the mutant and wild-type strains decreased when the broth viscosity increased because of gellan accumulation. The gellan production of the Δ*phaC*-Δ*crtI*–m10 mutant was 13% higher than that of the wild-type strain. The similar sucrose consumption rates of both strains while the Δ*phaC*-Δ*crtI*–m10 mutant showed higher gellan production may be attributed that more carbon originally used to produce PHB was converted to gellan, as is evident in Fig. 3A. Moreover, the PHB accumulation in ATCC 31461 strain is also involved in the high CDW (Fig. 3B). The yield increase observed for the Δ*phaC*-Δ*crtI*–m10 mutant was lower than those previously reported by West (16) and Lobas et al. (17). A combination of conventional chemical mutagenesis and antibiotic resistance was used by West to isolate a mutant. Gellan production by the mutant strain was about twofold higher than its parent strain on glucose after 48 h of growth (16). A new screening technique was used by Lobas et al. (17) to isolate the mutant strain (DSM 6314). The productivity was found to be about four times higher than that of the parent strain. However, a final gellan yield of 15.43 g liter^−1^ for the Δ*phaC*-Δ*crtI*–m10 mutant is higher than that of the two mutants mentioned above (11.3 g liter^−1^ and 9.8 g liter^−1^, respectively). The gellan yield is even higher than the result described by Banik et al. (18), in which the gellan production was 13.8 g liter^−1^ after the optimization of nutrients in response to the surface methodology. Generally, the pyruvate can be exported out of the cell through the cotransport of hydrogen ions with a special proton pump. Moreover, the conversion of sucrose to gellan by the Δ*phaC*-Δ*crtI*–m10 mutant was 60%, which was relatively high compared with low sugar conversion (40 to 50%) for most of the gellan production (19) (Table 3). The increase in gellan production and conversion might be the increase in gellan production and conversion resulting from more carbon flux in the direction of gellan gum synthesis for the carotenoid-PHB-free double gene knockout mutant strain. To further increase the gellan production, additional improvements in industrial fed-batch fermentation and optimization of culture conditions through use of the *phaC*-*crtI*−m10 mutant should be attempted in future studies.

**TABLE 3:** Comparison of existing strains and other gellan-producing strains^*a*^

Strain	Pyruvate level (mg liter^−1^)	Carotenoid level (mg liter^−1^)	PHB level (g liter^−1^)	Gellan yield (g liter^−1^)	Conversion rate of sucrose to gellan (%)	Reference
Δ*crtI*-*phaC*-2	500.2	NA	NA	5.6	37	This study
Δ*phaC*-Δ*crtI*−m10	249.3	NA	NA	15.4	60	This study
ATCC 31461	248.7	14.5	3.15	11.8	43	This study
EGP-1	Unknown	Unknown	Unknown	11.3	45	16
DSM 6314	Unknown	Unknown	Unknown	9.8	42	17

a All experimental data were means of triplicate measurements. NA, not available.

The production of gellan gum is, in general, a highly viscous microbial fermentation process, which leads to difficulties in gellan gum recovery (19). Moreover, the carotenoids and PHB are closely bound together with gellan gum, also making it difficult to recover the gellan. Accordingly, large amounts of ethanol are required in the conventional recovery method to dilute the fermentation broth (19), precipitate the biopolymer, and remove the carotenoid pigments. Moreover, the diatomite plate and frame pressure filtration to remove PHB consumes more human and material resources. Consequently, the significantly high cost of the organic solvents and complicated processing made gellan production economically unfavorable. A mutant strain that is deficient in carotenoid and PHB shows potential for a more economic downstream purification process. As a consequence, based on this high yield and the mutant producing gellan with higher purity, a new purification method that dispensed with the normal process steps for eliminating the carotenoid and PHB with less solvent (30% versus 200%) and fewer operating steps was developed to recover gellan from the broth. Moreover, higher gellan production would further reduce production costs and make it a promising strain for large-scale manufacturing needs. This method will effectively reduce the production costs and improve the economic benefits of large-scale gellan production.

## MATERIALS AND METHODS

### Strains, plasmids, and media.

The strains and plasmids used in this study are listed and described in Table 4. Unless indicated otherwise, the wild-type strains of Sphingomonas elodea ATCC 31461 (10) and its gene knockout mutants were cultured in yeast extract peptone-glucose (YPG) medium (30 g liter^−1^ yeast extract, 50 g liter^−1^ peptone, and 200 g liter^−1^ glucose [wt/vol for all ingredients]) at 30°C for 72 h and stored at 4°C. All Escherichia coli strains were cultured aerobically in LB medium on a shaker at 200 rpm and 37°C (20). The preculture medium for S. elodea contained 10 g liter^−1^ NaCl, 10 g liter^−1^ peptone, 5 g liter^−1^ yeast extract, and 5 g liter^−1^ sucrose. The gellan fermentation medium consisted of the following: 15 g liter^−1^ K_2_HPO_4_, 15 g liter^−1^ KH_2_PO_4_, 5 g liter^−1^ MgSO_4_·7H_2_O, 5 g liter^−1^ yeast extract, 30 g liter^−1^ sucrose, and 5 g liter^−1^ soy protein. The inoculum was placed in 250-ml Erlenmeyer flasks containing 50 ml of the preculture medium, which were incubated at 30°C and 220 rpm for 24 h. For fermentation, 10% inoculum was inoculated in 500-ml Erlenmeyer flasks and incubated on a rotary shaker at 30°C and 220 rpm for 48 h. The pH of all the media was adjusted to 7.2 (±0.1) by adding 1 M NaOH or 1 M HCl before sterilization. When required, the following antibiotics were used at the indicated concentrations: streptomycin (25 μg ml^−1^), kanamycin (50 μg ml^−1^), ampicillin (15 μg ml^−1^ for *Sphingomonas*; 100 μg ml^−1^ for E. coli), and tetracycline (5 μg ml^−1^ for *Sphingomonas*; 25 μg ml^−1^ for E. coli).

**TABLE 4:** Strains and plasmids used in this study

Strain or plasmid	Description or relevant genotype and/or phenotype	Source or reference(s)
S. elodea		
ATCC 31461	Wild type	ATCC
Δ*crtI*	*crtI* gene knockout mutant derived from ATCC 31461	This study
Δ*phaC*	*phaC* gene knockout mutant derived from ATCC 31461	This study
E. coli		
DH5α	F^−^ ϕ80d*lacZ*ΔM15 Δ(*lacZYA argF*)*U169 deoR recA1 endA1 hsdR17*(r_K_^−^ m_K_^+^) *pho supE44* λ^−^ *thi-1 gyrA96 relA1*	Lab collection
S17-1 λpir	*recA pro hsdR* RP4-2-Tc::Mu-Km::Tn*7*,λ-pir; mobilizer strain	20
HB101/pRK2013	HB101 harboring pRK2013; Km^r^	28
Plasmids		
pRK2013	ColE1 *mob* + *tra*_RK2_Δ*rep*_RK2_*repE*^−^ Km^r^	21, 28
pLO_3_	Tc^r^ *sacB*, RP4 *oriT*, ColE1 *ori*	21
pLO_3_-*crt*I	pLO_3_ carrying upstream 555 bp and downstream 699 bp of *crtI*	This study
pLO_3_-*pha*C	pLO_3_ carrying upstream 604 bp and downstream 805 bp of *phaC*	This study

### Construction of the Δ*crtI* mutant.

All primers used in this work are listed in Table 5. To construct the *crtI* gene knockout mutant, the suicide vector pLO_3_ provided by O. Lenz was used (21). The adjacent upstream flanking sequences (555 bp) were amplified with primers *crt*I-UF and *crt*I-UR, while primers *crt*I-DF and *crt*I-DR were used for amplifying the adjacent downstream flanking sequences (699 bp) (GenBank accession no. JN224892.1). The primers used to amplify the flanking fragments of the target genes contained SacI, XbaI, or PstI restriction site. The PCR products were digested and ligated to pLO_3_ to construct the recombinant plasmid pLO_3_-*crt*I, which was then introduced into E. coli S17-1, and the resultant strain was named E. coli S17-1/pLO_3_-*crt*I. Recombinant DNA techniques were performed by standard methods (22) or as instructed by suppliers.

**TABLE 5:** Primers used in this study

Primer	Primer sequence (5′ to 3′)^*a*^	Note
*crt*I-UF	AGTGAGCTCCGAGGACACCTATTACAG (SacI)	For amplifying the 555-bp upstream homologous sequence of *crtI*
*crt*I-UR	TATCTAGAGCGCATCAGCGGCTCCAG (XbaI)	
*crt*I-DF	CGTCTAGATTGGCGTGAACATCCAAGCC (XbaI)	For amplifying the 699-bp downstream homologous sequence of *crtI*
*crt*I-DR	GACTGCAGAAGCCGACCTTGCCCATAT (PstI)	
		
*pha*C-UF	GTCGGAGCTCTCAACCGCTTCTACATTCTC (SacI)	For amplifying the 604-bp upstream homologous sequence of *phaC*
*pha*C-UR	GCAGTCTAGAGGCGCGATCAGCTTGTTGTC (XbaI)	
*pha*C-DF	GGTCTCTAGATGGACTGGTTGGTTGCGT (XbaI)	For amplifying the 805-bp upstream homologous sequence of *phaC*
*pha*C-DR	TAATGCATGCCGACGACAGGCCCTTCAG (PstI)	
		
*sac*B-F	CGAACCAAAAGCCATATAAG	Used to test integration of the first-round recombinant
*sac*B-R	AGCGAAGTGTGAGTAAGTAA	
*crt*I-TF	GTCTATTGCCTGCCGTTC	Used to test the type of the strains for second crossover deletion of *crtI*
*crt*I-TR	GGCTGATAGCGTGTTTTC	
*pha*C-TF	CCGCTGTACGAACTGATCCA	Used to test the type of the strains for second crossover deletion of *phaC*
*pha*C-TR	CGTCGTCTTAGGTCCTTTGCT	

a The underlined sequences are restriction enzyme sites (shown in parentheses).

With the help of E. coli HB101/pRK2013 strains, the plasmid pLO_3_-*crt*I from E. coli S17-1/pLO3-pLO_3_-*crt*I to *S. elodea* was transferred by triparental filter mating (23). The triparental filter mating method was performed as described previously (24). The Δ*crtI* mutants were detected by selection for sucrose (8%) tolerance due to loss of the *sacB* gene on pLO_3_ (25), followed by PCR screening for those with the correct excision for crossover deletion. Diagnostic PCR, using primer pair *sac*B-F and *sac*B-R and primer pair *crt*I-TF and *crt*I-TR, was used to confirm each constructed strain.

### Construction of the Δ*crtI*-*phaC* mutant.

A carotenoid-deficient Δ*crtI* mutant was used as the original strain to further reconstruct the *phaC* gene (NCBI reference sequence NZ_AGFU01000055.1) knockout mutant. The upstream and downstream flanking sequences of the *phaC* gene were amplified by PCR with the primers shown in Table 5. The recombination, translation, isolation, and confirmation processes were conducted by the same methods as those of the construction of the Δ*crtI* mutant except for the use of different primers.

### Combined UV and ethylmethane sulfonate mutagenesis.

To enhance the gellan production of the gene knockout mutant, combined UV irradiation and ethyl methanesulfonate (EMS) mutagenesis were performed by the previously described method (8). Exponentially grown Δ*crtI*-*phaC* mutant cells containing approximately 3 × 10^8^ cells/ml were treated with 1% (vol/vol) EMS at 30°C for 60 min without agitation, followed by UV irradiation (30 W, 30-cm distance) for 45 s under magnetic stirring. The cells were then spread onto YPG agar plates and cultured for 30°C after appropriate dilutions. After 72 h, the colonies were randomly chosen and checked for gellan production in 500-ml Erlenmeyer flasks at 30°C and shaken at 200 rpm for 48 h.

### Genetic stability of the mutants.

The genetic stability of the mutants was determined by a continuous passage experiment. Mutants were continuously inoculated into fresh preculture medium at 30°C and 220 rpm for 24 h. Subsequent batch fermentations of strain ATCC 31461 and mutant were studied to analyze their gellan production and viscosity.

### Batch fermentation of ATCC 31461 and mutant in a 6.7-liter bioreactor.

Batch fermentations were studied in a 6.7-liter stirred bioreactor (BioSCADALab R’ALF plus, Bioengineering AG, Switzerland) with a 4.5-liter working volume. The process set points were as follows: pH, 7.0 (±0.1); temperature, 30°C; agitation rate, 400 rpm; and aeration rate, 1 vvm (volume of air and volume of medium per minute). Both agitation and aeration were kept constant throughout the process. The pH was controlled by the automatic addition of 3 M NaOH and 3 M HCl.

### Purification of gellan.

To extract and purify the gellan gum, broths were heated at 95°C for 30 min to kill the cells and deactivate the enzymes. The broths were then processed with lysozyme (20,000 U liter^−1^) and protease (100,000 U liter^−1^) at 37°C for 2 h to degrade the solid cellular debris. After that, 2 volumes of 99% ethanol was added to the broth of ATCC 31461 strain to remove pigment and precipitate gellan as previously described (18). Finally, the gellan was redissolved with the appropriate amount of water and then filtered in a plate frame pressure filtration to remove the small insoluble particles of PHB, and gellan was recovered. Alternatively, the broth of the Δ*crtI*-*phaC*−m10 mutant after enzyme treatment was mixed with 10 g liter^−1^ CaCl_2_ solutions followed by precipitation with 30% ethanol (vol/vol) instead of 2 volumes of 99% alcohol and plate frame pressure filtration.

### Analysis methods.

For gellan extraction, the fermentation broth was diluted with distilled water, heated for 15 min in a boiling water bath, and centrifuged at 15,000 rpm for 45 min at 25°C (2). Then, the sample was suction filtered to separate the supernatant and cell pellet. The cell pellet was dried to a constant weight at 60°C to measure cell dry weight (CDW). The supernatant was then added with absolute ethanol at a volume ratio of 1:3, followed by vigorous mixing and kept at 4°C overnight. After several washes with ethanol, the polymer was separated by centrifugation at 10,000 rpm for 45 min. Determination of the amount of gellan was accomplished by measuring the dry weight of the polymer recovered from the culture medium (26). The total sucrose content in the broth was estimated by the Fehling method after acid hydrolysis with 1 M HCl at 75°C for 10 min (8). The viscosity of the culture broth was determined at 25°C using a Brookfield viscometer model RVDV-II＋P (no. 4 spindle at 60 rpm). To determine the PHB content, the broth was treated with 5.75% sodium hypochlorite (5 ml) at 37°C for 16 h, and then concentrated sulfuric acid (4.8 ml) was added. The mixture was heated (100°C for 10 min), and the precipitate was separated by centrifugation. The PHB content was then quantitatively analyzed using gas chromatography after methanolysis of lyophilized cells in chloroform. The total carotenoid content of the fermentation broth was determined spectrophotometrically as described previously (27). The pyruvate content of the broth was measured by the method described previously (8).

### Data availability.

The *phaC* and *crtI* sequences have been deposited in NCBI under accession numbers NC_009511.1 and NZ_BCTR01000055.1, respectively.
