# Supplementary figures and images for: A Carotenoid- and Poly-β-Hydroxybutyrate-Free Mutant Strain of Sphingomonas elodea ATCC 31461 for the Commercial Production of Gellan
Source: mSphere. 2019 Oct 16;4(5):e00668-19. doi: 10.1128/mSphere.00668-19 (PMC6796983; doi:10.1128/mSphere.00668-19)

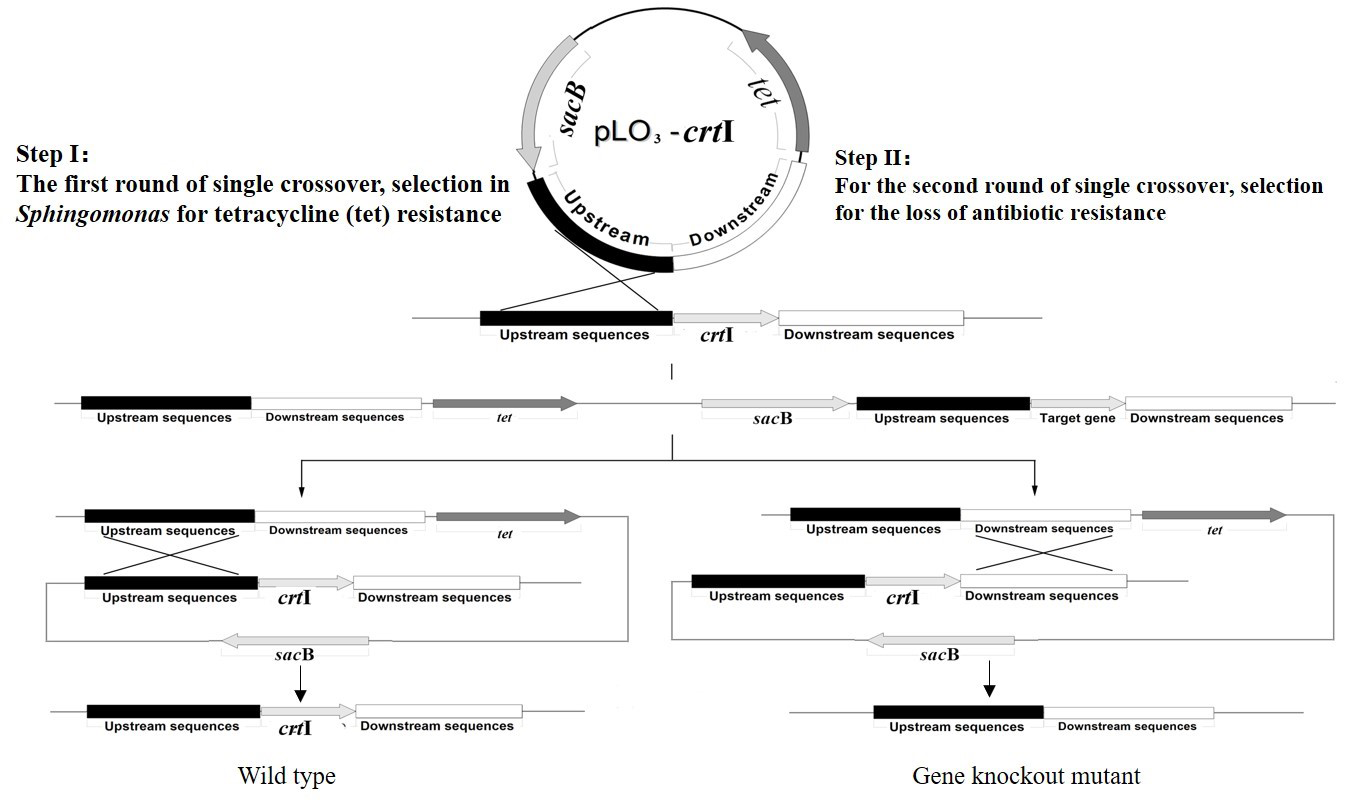

Supplement: FIG S1 [file mSphere.00668-19-sf001.tif]

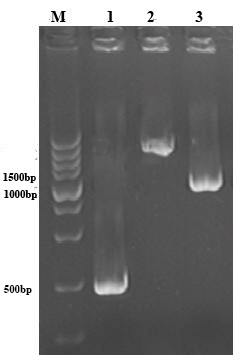

Supplement: FIG S2 [file mSphere.00668-19-sf002.tif]

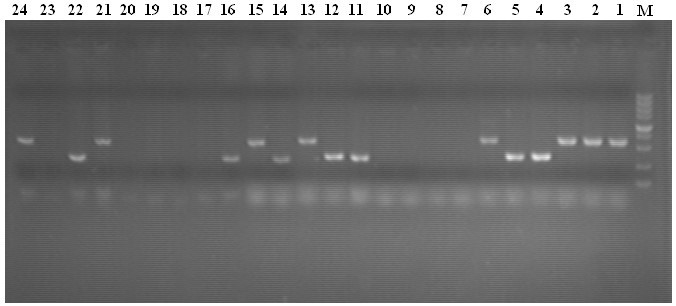

Supplement: FIG S3 [file mSphere.00668-19-sf003.tif]
